# A wireless buckle transducer for measurement of human forearm tendon tension: operational principles and finite element study

**DOI:** 10.3389/fbioe.2024.1278740

**Published:** 2024-11-27

**Authors:** Alireza Rastegarpanah, Stephen J. G. Taylor

**Affiliations:** ^1^ School of Metallurgy and Materials, University of Birmingham, Birmingham, United Kingdom; ^2^ Division of Surgery and Interventional Science, Royal National Orthopaedic Hospital, Institute of Orthopaedics and Musculoskeletal Science, University College London, Stanmore, United Kingdom

**Keywords:** buckle transducer, tendon, calibration, regression, finite element analysis

## Abstract

**Introduction:**

Conventional methods for evaluating the management of spasticity, a complex neuromuscular disorder, typically fail to directly measure the muscle forces and loads applied through tendons, which is crucial for accurate diagnostics and treatment. To bridge this gap, we developed a novel modular buckle transducer (BT) designed to measure tendon forces *in vivo*. This device adjusts to accommodate tendon sizes ranging from 3 mm to 5 mm, maintaining accuracy within this range and avoiding the need for identical tendon calibration.

**Methods:**

This study first presents the mechanical principles for determining tendon tension T using several strain gauges appropriately positioned to allow for varying angles of passage of the tendon through the device. Next, we present a finite element (FE) model that uses multiple linear regression to determine T while varying tendon diameter and lateral placement within the device for several candidate strain gauge locations on the device base plate. Finally, we posit several alternative ways of combining gauge strains.

**Results:**

Initial simulation results demonstrated that this placement facilitates effective pre-implementation calibration, with the device accommodating tendon variations from 3 mm to 5 mm in diameter for a range of gauge placements.

**Discussion:**

Future validation of this technology will involve direct testing on explanted human/equine tendons to verify the practical utility of the BT, aiming to establish a new standard for assessing and managing neuromuscular disorders such as spasticity.

## 1 Introduction

Spasticity is a motor disorder arising from anomalies in the brain or spinal cord, manifesting through increased muscle tone and exaggerated tendon jerks due to neurophysiological changes Mukherjee and Chakravarty (2010). Tendons serve as essential conduits, transmitting muscle forces to bones; the tensile force transmitted via tendons may be determined using both invasive and non-invasive techniques. Invasive methods, such as the direct implantation of force transducers, pose risks such as infection, signal loss, and moisture ingress, making them less favourable for long-term applications. On the other hand, non-invasive techniques, while safer, often lack the precision required for dynamic, *in vivo* measurements of tendon forces. The buckle transducer (BT) bridges this gap by offering a minimally invasive solution that enables accurate and direct tendon force measurements.

In recent years, non-invasive methods for tendon force measurement have been proposed, utilizing technologies such as ultrasound and shear wave propagation. Pourcelot et al. demonstrated that tendon forces can be non-invasively estimated by analyzing the propagation of ultrasonic waves along the tendon Pourcelot et al. (2005). Additionally, a shear wave tensiometer introduced by Martin et al. uses skin-mounted sensors to measure tendon vibrations, providing a non-invasive method for estimating tendon loads during dynamic activities such as walking and running Martin et al. (2018). While these methods offer valuable alternatives, they are typically limited to superficial tendons and rely on optimal acoustic coupling with the skin for accuracy. Furthermore, they may not provide the precision required for more complex, *in vivo* measurements, especially in deeper tendons. In contrast, buckle transducers (BT) provide a direct and highly accurate measurement of tendon forces, particularly in scenarios where non-invasive methods may fall short due to tendon depth or the need for precise, dynamic measurements.

While natural asymmetry between the left and right limbs, such as differences in muscle mass, tendon length, and joint structure, may impact tendon force transmission, this study focuses on measuring the force within the specific tendon into which the transducer is inserted. Our goal is to provide accurate measurements for the targeted tendon, irrespective of potential asymmetry between limbs. Among these techniques, buckle transducers (BTs) are notable for their ability to measure forces directly by placing the BT around the tendon such that it senses the force applied to it by the tendon without injury. These BTs, classified under extensometry transducers, measure the *in vivo* force exerted by tendons, with strain gauges embedded in the device to reflect these forces Ravary et al. (2004).

Buckle transducers function by utilizing the natural tendency of a tendon, when intertwined between the deformable arms of the device, to straighten upon tensile loading. As the tendon straightens, it exerts a force that deforms the transducer’s arms. This deformation is captured by strain gauges mounted on the device, which convert the physical strain into electrical signals for analysis. This method allows for the measurement of forces within the tendon without causing tissue damage, making BTs suitable for *in vivo* applications.

**FIGURE 1:**
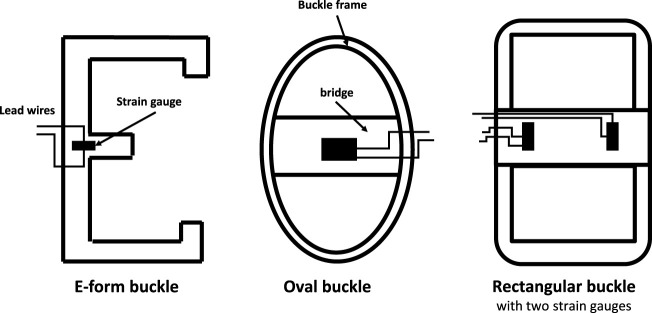
Some alternative designs of buckle transducer in the literature, with sectional view Keegan et al. (1991); Roberts (1994); Stone et al. (1983); the E-form (and its variant, the S-form) allow buckle insertion and removal *in vivo*; others require a removable crossbar Ravary et al. (2004).

Various BT designs have been developed over the years, including the E-form, oval, and rectangular form configurations (Figure 1), each suited to different biomechanical applications. The E-form design, for example, offers ease of insertion and removal, making it ideal for *in vivo* applications. The oval and rectangular form configurations provide greater contact area with the tendon, which can enhance measurement sensitivity but may require more complex calibration. These designs represent alternative approaches to tendon tension measurement, rather than an evolutionary progression.

This study introduces a modular buckle transducer (BT) designed to accommodate various Flexor Carpi Radialis tendon sizes. We hypothesize that the BT can be effectively calibrated across different tendon thicknesses and tension loads, particularly in vitro environments. Our approach involved mathematical modelling followed by Solidworks simulations to record the impact of varying forces on the BT using multiple bearing sizes. These simulations were instrumental in determining the optimal locations for installing gauge sensors on the buckle’s baseplate, aiming to maximise accuracy.

### 1.1 Buckle transducers reported in human studies

Buckle Transducers (BTs) have garnered extensive attention due to their instrumental significance in diagnosing tendon issues, facilitating treatment, and investigating the biomechanics of tendons and ligaments. In human studies, BTs find wide applicability in assessing Achilles tendon force, a pivotal element in normal walking. The anatomical location of the Achilles tendon allows for safe implementation of the BT around the tendon, minimizing risks to surrounding tissues Gregor et al. (1987), Fukashiro et al. (1993), Komi (1990). In one study, a small S-shaped BT was implanted in subjects with spasticity to measure the force of the knee’s distal spastic semitendinosus tendon Ateş et al. (2016). Another research endeavor employed a BT to measure loads on knee ligaments during knee flexion, contributing to the validation of a reconstruction technique Coobs et al. (2010). Furthermore, a custom-designed rectangular BT was utilized for *in vitro* testing, enabling the study of intact ankle ligament properties at varying loads using a hydraulic material testing machine Best et al. (2016). These diverse applications underscore the versatility and significance of BTs in biomechanical research and clinical investigations.

Indeed, BTs have also found application in upper-limb studies. For instance, a study reported *in vivo* measurement of hand tendon forces using an S-shaped force transducer Schuind et al. (1992). In another study, a BT was implanted in the tendons of the index finger to measure flexor tendon forces Edsfeldt et al. (2015). Furthermore, a strain gauge-based BT was employed to measure tension in wrist flexor tendons Fridén et al. (2010). These investigations underscore the adaptability and utility of BTs across various upper-limb scenarios, contributing valuable insights into the biomechanics and function of tendons in these regions.

### 1.2 Buckle transducers reported in animal studies

In animal studies, BTs have been applied to diverse contexts. For instance, an E-shaped stainless steel BT equipped with a metal foil strain gauge was employed to measure Achilles tendon force in a goat Lee et al. (2011). In another investigation involving horses, an E-type BT was implanted in eight subjects to gauge the force required for optimal intraoperative left arytenoid cartilage abduction, aiding in laryngoplasty surgery Witte et al. (2010b). Transducer calibration, conducted before and after implantation, served to validate transducer sensitivity, with linear regression establishing the correlation between force and voltage output. Another E-type transducer was developed to measure forces exerted on surgical sutures Witte et al. (2010a). Regression measurement yielded predictions of actual force with a mean error of 4%, deemed an acceptable prediction margin for *in-vitro* calibration.

In a separate study, the performance characteristics of an internal BT were evaluated analytically *in vivo* Herzog et al. (1996). The findings revealed that even minor angular displacements and corresponding bone rotations could significantly influence transducer output. While internal force transducers do produce reliable signals *in vivo*, precise determination of tissue forces can prove challenging, especially during animal locomotion. These varied applications highlight the value of BTs in animal biomechanical research, offering insights into physiological processes and aiding surgical interventions.

### 1.3 Sizes of BT and associated risks of implantation

The sizes of implanted BTs have been tailored to match the dimensions of the respective tendons. For instance, in animal studies, an implantable E-form BT was reported to have dimensions of approximately $9\times 5mm^{2}$ Wehrle et al. (2001), while in human studies, dimensions were around 34×20mm Ravary et al. (2004). In another example, an S-shape BT measuring 20×12× 9 mm with a maximal force range of 400 N was employed to measure the force exerted on human spastic muscles during knee flexion Yucesoy et al. (2017). These diverse sizes reflect the adaptability of BTs to varying anatomical requirements and research objectives, making them a versatile tool in biomechanical investigations.

Long-term implantation of BTs in the body can lead to notable discomfort, particularly when the transducers are bulky. A study found that pain levels were significantly alleviated upon reducing the size of the BT Komi et al. (1987). The duration required for wound healing and the return to normal walking after BT implantation varied, ranging from 2 to 3 weeks in humans Ravary et al. (2004), to 1 week in cats Walmsley et al. (1978). Two potential risks associated with BT implantation include tissue shortening and bone impingement, both of which can be mitigated by employing smaller BTs. Each application often demands a specific BT geometry tailored to the unique requirements of the study.

A primary advantage of BTs lies in their capacity to offer a mechanical average of force, reducing errors stemming from unmeasured sections of the tendon Fleming and Beynnon (2004). However, even slight misalignment of the transducer’s axis relative to the axis of tendon fibers can induce significant fluctuations in the output signal for a given externally applied load Ravary et al. (2004). This underscores the importance of meticulous positioning and alignment of BTs to ensure accurate and reliable measurements.

### 1.4 Calibration issues

Similar to any measurement device used in inaccessible environments, the accuracy of *in vivo* readings obtained from BTs relies on dependable and reproducible calibration, either before or after the experiment. In the case of permanently implanted devices, particularly in humans, calibration must be conducted beforehand using materials that closely resemble the tendon’s properties. However, this task is challenging due to the non-homogeneous nature of tendons and the complexities of replicating their characteristics outside the body within a calibration setting. Instances in the literature highlight situations where inadequate calibration, failing to account for local movement or shape changes of the tendon *in vivo*, resulted in unreliable measurements. Typically, calibration involves clamping the ends of a substitute material mimicking the tendon on either side of the transducer within a tensile testing machine. Ideally, the substitute material should have similar properties and geometry to the actual tendon. However, in cases where the transducer’s sensitivity to changes in tendon geometry comes into play, this calibration approach proves inadequate due to unknown morphological changes occurring with load. Hence, calibration is a critical step that requires careful consideration and adaptation to account for the intricacies of the biomechanical context. Addressing the challenges associated with proper calibration ensures that the measurements obtained from BTs accurately reflect the forces within tendons and support meaningful interpretations of biomechanical phenomena.

Interpreting the data obtained post-calibration presents challenges due to the potential influence of local variations in tendon geometry on the relationship established between the transducer signal and tendon load during calibration. It becomes evident that *in vitro* calibration necessitates independent validation methods to accurately align with the *in vivo* conditions Ravary et al. (2004). For example, a study by An et al. An et al. (1990) developed a low-profile transducer equipped with two uniaxial strain gauges to measure loads ranging from 0 to 50 N. The findings indicated that repeated loading and the consequent alterations in tendon thickness have an impact on the calibration factor. Additionally, tendon thickness emerged as a parameter that could be leveraged to affect the calibration factor. These complexities emphasize the significance of comprehensive calibration procedures that consider the intricate interplay between transducer signals, tendon geometry, and load. Addressing these challenges ensures that the collected data accurately reflects the biomechanical dynamics within tendons and supports robust and meaningful interpretations of the results.

It is evident that prior research, whether conducted in humans or animals, has predominantly relied on either post-calibration of tendons (in the case of animals) or attempting to estimate *in vivo* tendon thickness while tolerating uncertainties about the forces by calibrating with materials of similar geometry. In this context, the primary contribution of this paper lies in presenting a proof-of-concept for a modular BT design that overcomes the limitations of conventional approaches. This novel design enables the measurement of tendon tension in a manner that is independent of the tendon’s cross-sectional area and its position within the BT, using multiple strain gauges to avoid the need for pre- or post-calibration using the target tendon.

## 2 Methodology

**FIGURE 2:**
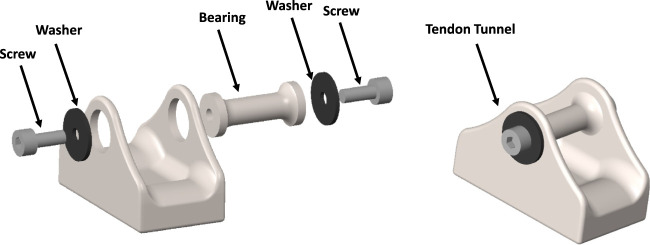
Designed CAD model of the modular buckle transducer in disassembled view (left) and assembled view (right).

This study introduces a novel design for a BT featuring a detachable central bearing, as depicted in Figure 2. The integration of both measurement and data telemetry capabilities within a single hermetically sealed unit presented several challenges. These included maintaining strain sensitivity (which required the use of temperature-sensitive silicon strain gauges), allocating sufficient space for the amplifiers, analog-to-digital converters, and telemetry circuitry, and ensuring the hermetic seal remained intact to protect the electronics from environmental factors. Additionally, the design had to allow for effective near-field coil coupling, using the same pair of coils for both power supply and data telemetry. While these challenges were addressed in the design, further optimization, especially concerning strain sensitivity, remains a potential area for improvement.

This study currently restricts itself to a finite element, and while the design is optimized to accommodate these components, no *in vivo* testing has yet been conducted. The integration of power supply and telemetry circuitry remains theoretical at this stage. An implanted coil within the device would enable inductive coupling with an external coil, serving the dual purpose of power supply and data telemetry. This innovative approach aligns with the pursuit of streamlined and integrated solutions for tendon force measurement and data transmission.

### 2.1 Design of modular buckle transducer

The study at hand focuses on the modelling of a BT with dimensions of 20 × 11 × 13 mm (Length-Width-Height). This design incorporates a cavity on the underside of the transducer to accommodate strain gauges, electronics, and an induction coil. The selected dimensions are in practical alignment with the feasibility of manufacturing such a transducer. Notably, the BT’s design has been tailored for surgical convenience, enabling the central bearing to be removed and subsequently repositioned over the tendon. This feature facilitates the passage of the tendon through the transducer, with bearing surfaces present at both ends and at the central bearing. This design approach prioritizes usability during surgical procedures while maintaining the essential functionalities of the transducer.

As the tendon tension increases, the natural response of the tendon is to attempt to straighten. This action places direct mechanical load on the three positioned bearings within the BT. Depending upon the tendon’s diameter, it will be deflected through the BT with a varying small angle. This will impose a combination of axial and shear forces on the end bearings, which will vary the proportion of strain in gauges located at different distances from the centre. The challenge is to interpret these strains to measure the tensile force in the tendon, independent of the tendon position and shape.

A study by Weber et al. Weber et al. (2015) measured the cross-section of the FCR tendon across multiple specimens, revealing diameter variations ranging from 3mm to 5 mm. Correspondingly, our BT was designed to feature six distinct removable rod sizes, specifically 3 mm, 3.4 mm, 3.8 mm, 4.2 mm, 4.6 mm, and 5 mm, which correspond to the typical tendon sizes encountered in the tendons of interest (Figure 4). The current design has not been tested on tendons outside this range. If applied to tendons significantly larger or smaller than these sizes, the measurement accuracy could be affected. Expanding the central rod size range would require further calibration and testing to ensure accuracy across a broader range of tendon sizes. This array of sizes empowers surgeons to select the optimal bearing size during surgery, effectively ensuring a suitable deflection angle (angle of passage) that enables the achievement of a desirable strain magnitude at the strain gauges. Importantly, this approach prevents overstretching of the tendon while maintaining the capacity to generate adequate strain magnitudes.

### 2.2 Mathematical modelling of buckle transducer

**FIGURE 3:**
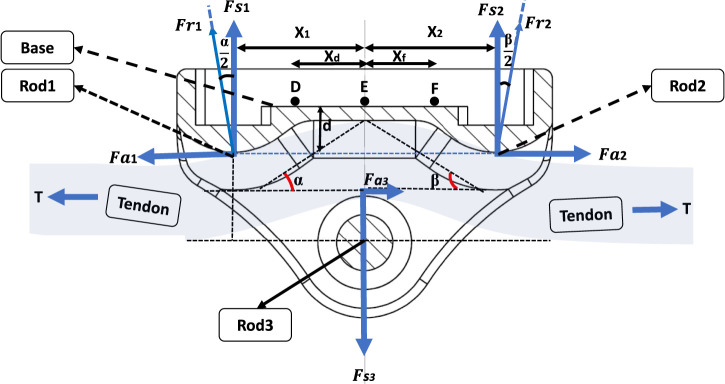
Free body diagram of the designed buckle transducer in cross-section view.

A 2D mechanical analysis was carried out to establish relationships between strains at candidate strain gauge sites on the BT’s baseplate, the tension within the tendon (T), and the angles of passage ( α , β ) on either side of the transducer (Figure 3). This analytical investigation treated the BT as an unconstrained frictionless free body. It was reasonably assumed that the BT is effectively rigid compared to the tendon and the loads generated. The analysis considered force vectors acting on the rods, which arise from the magnitude of T and the two angles of passage ( α , β ). The alteration in the tendon’s direction around each bearing surface gives rise to two orthogonal forces at each bearing, the vertical (shear - $F_{s}$ ) and horizontal (axial - $F_{a}$ ) components of the resultant force $\left(F_{r}\right)$ acting on bearing one are given by Equations 1a-c:

$$F_{a1}=F_{r1}\sin\left(\frac{\alpha }{2}\right)$$ (1a)

$$F_{s1}=F_{r1}\cos\left(\frac{\alpha }{2}\right)$$ (1b)

$$F_{r1}=2T\sin\left(\frac{\alpha }{2}\right)$$ (1c)

At bearing 2, given by Equations 2a-c:

$$F_{a2}=F_{r2}\sin\left(\frac{\beta }{2}\right)$$ (2a)

$$F_{s2}=F_{r2}\cos\left(\frac{\beta }{2}\right)$$ (2b)

$$F_{r2}=2T\sin\left(\frac{\beta }{2}\right)$$ (2c)

Therefore, the relations between T and its orthogonal components are as follows:

At bearing 1, given by Equations 3a-b:

$$F_{a1}=2T\sin^{2}\left(\frac{\alpha }{2}\right)=T\left(1-\cos\alpha \right)$$ (3a)

$$F_{s1}=2T\cos\left(\frac{\alpha }{2}\right)\sin\left(\frac{\alpha }{2}\right)=T\sin\left(\alpha \right)$$ (3b)

At bearing 2, given by Equations 4a-b:

$$F_{a2}=2T\sin^{2}\left(\frac{\beta }{2}\right)=T\left(1-\cos\beta \right)$$ (4a)

$$F_{s2}=2T\cos\left(\frac{\beta }{2}\right)\sin\left(\frac{\beta }{2}\right)=T\sin\left(\beta \right)$$ (4b)

At bearing 3, given by Equations Equations 5a-b:

$$F_{s3}=Tsin\alpha +Tsin\beta$$ (5a)

$$F_{a3}=T\cos\beta -T\cos\alpha$$ (5b)

These forces lead to the generation of bending moments within the baseplate, along with slight axial forces. In order to differentiate the sensitivities to T, α , and β , a total of six separate strain gauges were simulated. One pair was located at each of the designated points (D, E, F) on the BT, one gauge per side of the buckle (Figure 3).

In the subsequent analysis, we consider the transducer to be in a state of static equilibrium around the tendon, characterized by the following conditions, given by Equations 6a-b:

$$F_{s1}+F_{s2}=F_{s3}$$ (6a)

$$F_{a2}+F_{a3}=F_{a1}$$ (6b)

Where the tendon is of uniform cross section throughout the BT, the buckle exhibits symmetry, and coupled with the absence of friction between the tendon and the bearings, the following relationships, Equations 7a-b, hold:

$$F_{s1}=F_{s2}=\frac{F_{s3}}{2}$$ (7a)

$$F_{a1}=F_{a2};F_{a3}=0;\alpha =\beta$$ (7b)

However, in more general scenarios, the tendon thickness does not remain constant, leading to α≠β . In such cases, there are three unknown variables (T, α , β ) that need to be determined. To establish the relationships between these variables and the three strains (or pairs of strains) at points D, E, and F, we derive three equations. The axial and shear forces applied to the buckle’s bearing surfaces due to the tendon induce components of strain. Treating the structure as a beam with simple supports at the end bearings and subject to a concentrated shear force $F_{s3}$ , we observe that no moments are sustained at the end bearings. Consequently, the bending moments at points D, E, and F can be expressed by Equations 8a-c:

$$M_{D}=F_{s1}\left(X_{1}-X_{d}\right)+\left(d\times F_{a1}\right)$$ (8a)

$$M_{E}=F_{s1}X_{1}+\left(d\times F_{a1}\right)$$ (8b)

$$M_{F}=F_{s2}\left(X_{2}-X_{f}\right)+\left(d\times F_{a2}\right)$$ (8c)

Where d is the perpendicular distance from the line of action of Fa1 , Fa2 to the strain gauged surface. Substituting T, α , β for the forces using Equations 3, 4 we find (Equations 9a-c):

$$M_{D}=T\sin\alpha \left(X_{1}-X_{d}\right)+dT\left(1-\cos\alpha \right)$$ (9a)

$$M_{E}=T\sin\alpha \times X_{1}+dT\left(1-\cos\alpha \right)=T\sin\beta \times X_{2}+dT\left(1-\cos\beta \right)$$ (9b)

$$M_{F}=T\sin\beta \left(X_{2}-X_{f}\right)+dT\left(1-\cos\beta \right)$$ (9c)

And using the relationships between bending and strain at each site, including tensile strains due to axial forces $F_{a_{1}}$ , $F_{a_{2}}$ , given by Equations 10a-d:

$$\varepsilon_{D}=\frac{\left(T\sin\alpha \left(X_{1}-X_{d}\right)+dT\left(1-\cos\alpha \right)\right)y_{D}}{I_{D}E}-\frac{T\left(1-\cos\alpha \right)}{A_{D}E}$$ (10a)

$$\varepsilon_{E}=\frac{\left(T\sin\alpha \times X_{1}+dT\left(1-\cos\alpha \right)\right)y_{D}}{I_{E}E}-\frac{T\left(1-\cos\alpha \right)}{A_{E}E}$$ (10b)

$$\varepsilon_{E}=\frac{\left(T\sin\beta \times X_{2}+dT\left(1-\cos\beta \right)\right)y_{E}}{I_{E}E}-\frac{T\left(1-\cos\beta \right)}{A_{E}E}$$ (10c)

$$\varepsilon_{F}=\frac{\left(T\sin\beta \left(X_{2}-X_{f}\right)+dT\left(1-\cos\beta \right)\right)y_{F}}{I_{F}E}-\frac{T\left(1-\cos\beta \right)}{A_{F}E}$$ (10d)

Where $y_{n}$ is the distance from the neutral axis to the gauge site, $I_{n}$ is the second moment of the area at each cross-section, and $A_{n}$ is the local cross-sectional area (n=D,E,F) where Yn, In and An are all known. These equations may be solved to determine T, α and β , independent of locally varying angles of passage. In practice, the sensitivity coefficients are found using multiple linear regression in pre-implantation calibration using a range of tendons or substitute materials over the diameter range 4–5 mm (with appropriate sized central rod).

### 2.3 Finite Element Analysis in simulation

**FIGURE 4:**
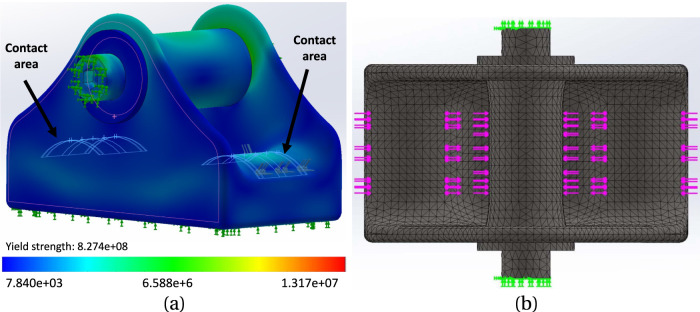
**(A)** The simulated model of the buckle, with 4.2 mm rod, under stress; the range of stress change is depicted by the colour spectrum. The contact areas demonstrate the locations where the tendon interacts with the buckle. **(B)** mesh model of the buckle with a global mesh size of 0.49 mm and tolerance of 0.02 mm.

To determine the optimal locations for the strain gauges within the practical transducer, the Finite Element Analysis (FEA) toolbox in Solidworks was employed. This analysis aimed to ascertain the strains generated along the baseplate of the modelled BT in response to the tendon tension, T, and the angles of passage, α and β . The purpose was to characterize the strain behaviour in terms of its sensitivity to T, as well as its response to changes in tendon diameter and its position within the buckle. For each tendon size, ranging from 4mm to 5 mm in diameter with 0.2 mm increments, a corresponding sized central rod was employed to accommodate the tendon with appropriate angles of passage. This ensured a consistent angle of passage (13°) across all tendon diameters, which represented a suitable angle for achieving adequate strain sensitivity. A range of five tensile loads, from 100N to 500N, was applied to assess linearity, while 500 N was used for all other variables tested. The vertical and horizontal components of the force, both in magnitude and direction, acting on the rods were calculated using Equations 3–5. These forces were then applied to the bearing surfaces as distributed loads. To simplify the analysis, the tendon itself was not explicitly modeled in the simulation. Rather, the force distribution applied to the contact surfaces between the buckle and the tendon were modelled as depicted in Figure 4.

### 2.4 Determining the best location for mounting the gauges on the baseplate

**FIGURE 5:**
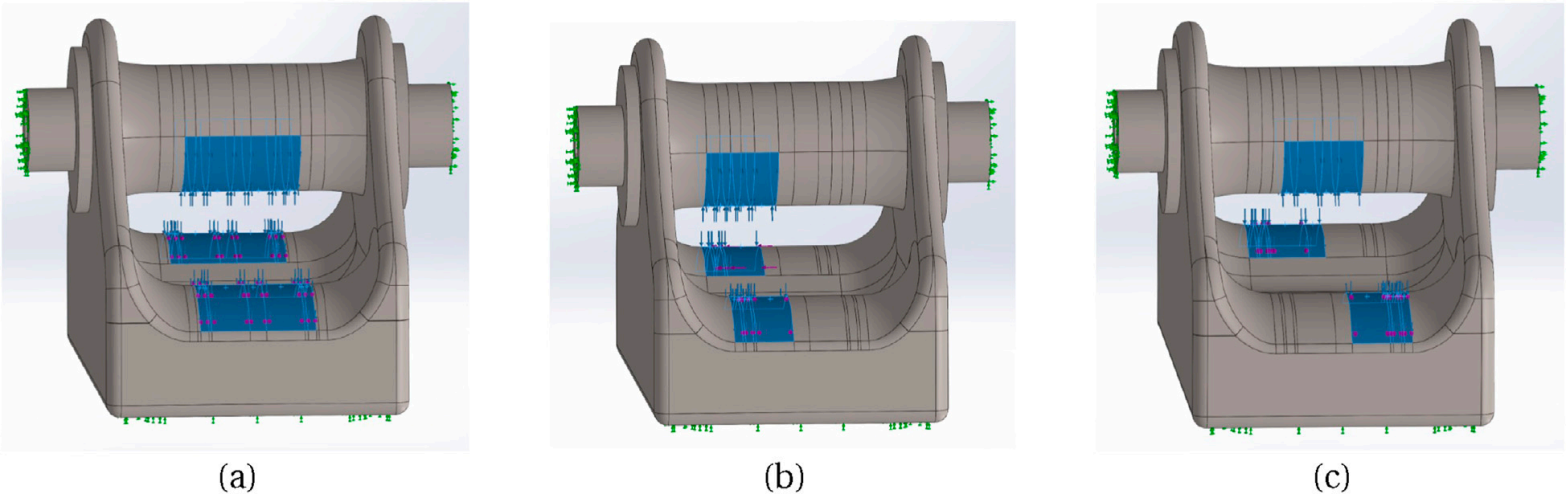
Three different tendon placement locations: **(A)** Centre, **(B)** Towards left side, **(C)** Diagonal.

Five alternative gauge placements were tested, each with six point strains measured from the FE model. In each case the six gauges were placed symmetrically about *X* and *Y*-axes and at X = 0, on the flat transducer baseplate; Table 1. The following load case sets were applied:

1. 100–500 N in 100 N increments for 4.4 mm tendon diameter, tendon centred;
2. 500 N for six increments of tendon diameter from 4 to 5 mm, tendon centred;
3. 500 N for 4.2 mm tendon diameter; three different positions of the tendon in the buckle: centred (Figure 5A), towards one side (Figure 5B), diagonal (Figure 5C). Extreme side and diagonal cases were also analysed (not shown).

**TABLE 1:** Location of sensors X, Y ( mm ).

Cases	Sensor 1	Sensor 2	Sensor 3	Sensor 4	Sensor 5	Sensor 6
1	(-1.9, 2.5)	(-1.9, −2.5)	(0, 2.5)	(0, −2.5)	(1.9, 2.5)	(1.9, −2.5)
2	(-1.9, 3)	(-1.9, −3)	(0, 3)	(0, −3)	(1.9, 3)	(1.9, −3)
3	(-2.9, 3)	(-2.9, −3)	(0, 3)	(0, −3)	(2.9, 3)	(2.9, −3)
4	(-4.1, 2.5)	(-4.1, −2.5)	(0, 2.5)	(0, −2.5)	(4.1, 2.5)	(4.1, −2.5)
5	(-4.1, 3)	(-4.1, −3)	(0, 3)	(0, −3)	(4.1, 3)	(4.1, −3)

**FIGURE 6:**
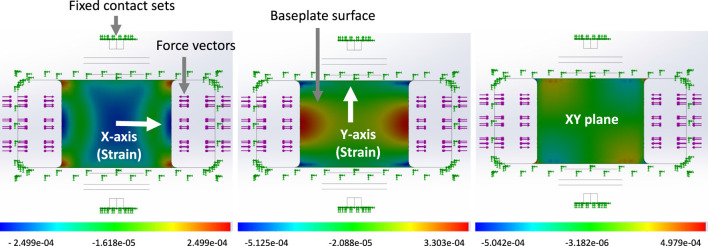
An example of Strain distribution on the baseplate when a 500N tension load was applied to the buckle with a 5 mm installed rod. The strain distribution is depicted from left to right: direct X, direct Y, XY shear. The figures display a bottom view of the buckle.

Figure 6 illustrates the distribution of X, Y direct strains and XY shear strain across the baseplate, for one load case of set 2, showing strain gradients along *X* and *Y*-axes. Table 2 lists three alternative ways of combining the six measured strains; these combinations were explored in the analysis, as potentially combining gauges electrically within a later experimental transducer would reduce the bandwidth requirements for data transmission. In practice the gauge locations would be determined by practical considerations as well as modelled results; strain gauges are a finite size and would have some tolerance on positioning, so by varying the locations in the model it is possible to ascertain how critical the experimental placements might have to be.

**TABLE 2:** Three alternative combinations of the 6 strains, referred to in table 3.

Strain combination	Strain values
1	$S=\left[\begin{array}{c} S_{1},S_{2},S_{3},S_{4},S_{5},S_{6} \end{array}\right]$
2	$S=\left[\begin{array}{c} \frac{\left(S_{1}+S_{2}\right)}{2},\frac{\left(S_{3}+S_{4}\right)}{2},\frac{\left(S_{5}+S_{6}\right)}{2} \end{array}\right]$
3	$S=\left[\begin{array}{c} \frac{\left(S_{1}+S_{2}+S_{5}+S_{6}\right)}{4},\frac{\left(S_{3}+S_{4}\right)}{2} \end{array}\right]$

### 2.5 Linear regression with least squares method

**FIGURE 7:**
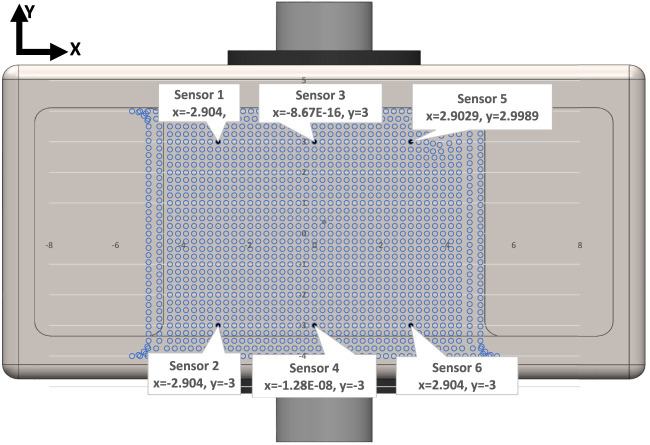
Schematic view of location of strain gauge sensors based on the positions provided by Case3 in Table 1. The blue dots show the location of the nodes on the baseplate surface.

Following the determination of sensor positions on the baseplate, linear regression using the least squares method was employed to compute the residual vector for both tendon load and diameter. The gathered dataset comprises n paired observations encompassing the independent variables X and the dependent variable Y . Consequently, the fitting model is given by Equation 11:

$$Y=\zeta_{0}+\zeta_{1}X+\epsilon$$ (11)

where ϵ represents the error vector, uncorrelated across measurements, while ζ signifies the parameter vector. In the present study, the applied tension load and tendon diameter serve as independent variables, with strain values as dependent variables. As indicated in Table 2, the strain matrix $S_{i,j}$ can adopt varying dimensions based on the predictor presentation format. We explore three distinct strain combinations relative to the strain gauge positions: (i) $S_{11,6}$ , where i∈1,2,…,11 signifies the number of observations, and j∈1,2,…,6 represents individual strain values calculated across six sensors; (ii) $S_{11,3}$ , where i∈1,2,…,11 signifies observations, and j∈1,2,3 represents three pairs of averaged strain values; (iii) $S_{11,2}$ , where i∈1,2,…,11 indicates observations, and j∈1,2 signifies averaged strain values for outer and inner sensors. The sensor locations, labeled one through 6, are illustrated in Figure 7.

In the analysis, we utilized Excel to conduct linear regression, facilitating the computation of coefficients $\zeta_{0}$ and $\zeta_{1}$ . The residual vector, denoted as e , is determined by Equation 12a. To ascertain the matrix error $e_{ij}$ —where i signifies the number of observations and j signifies the number of dependent variables—for the prediction of both tendon load and diameter, a structured approach was adopted. Initially, a matrix $A_{m\times n}$ was created, encompassing coefficients of dependent variables. In this context, m represents the count of dependent variables, while n denotes the number of independent variables. For instance, the residual error in predicting tendon tension and diameter, where strains are presented in the format of $S_{11,2}$ (corresponding to Strain combination two from Table 2), is given by Equations 12a, 12b:

$$e_{\left(i,1\right)}^{\mathrm{load}}={\left.{\left(A^{T}\times {\left(A\times A^{T}\right)}^{-1}\right)}^{T}\times S_{\left(i\times 3\right)}^{T}\right)}^{T}-L_{i},\;\;\;\;\;\;\;i\in \left\{1,2,\ldots ,11\right\}$$ (12a)

$$e_{\left(i,2\right)}^{\mathrm{Thickness}}={\left.{\left(A^{T}\times {\left(A\times A^{T}\right)}^{-1}\right)}^{T}\times S_{\left(i\times 3\right)}^{T}\right)}^{T}-Q_{i},\;\;\;\;\;\;\;i\in \left\{1,2,\ldots ,11\right\}$$ (12b)

Where L and Q represent the initial tension and diameter values respectively, and they were used as input in the regression model. As in some cases the matrices were non-square, the appropriate version of the Moore-Penrose pseudo inverse technique was used to make them invertible.

### 2.6 Effect of non-central location of the tendon

The initial study was devised around three key independent variables: the tendon’s diameter, the load it carried, and its position within the buckle. To examine scenarios where the tendon was not centrally seated within the buckle, three primary situations for tendon contact with the end bearings were simulated: i) *Central*: This situation entails the tendon aligning precisely along the buckle’s midline on the Y-axis. ii) *Diagonal*: Here, the tendon is positioned diagonally across the buckle. iii) *One-side*: This involves situating the tendon on one side (either right or left) of the bearing, in close proximity to a side wall. These represent possible extreme displacements, for which we still look for adequate measurement accuracy.

## 3 Results

**FIGURE 8:**
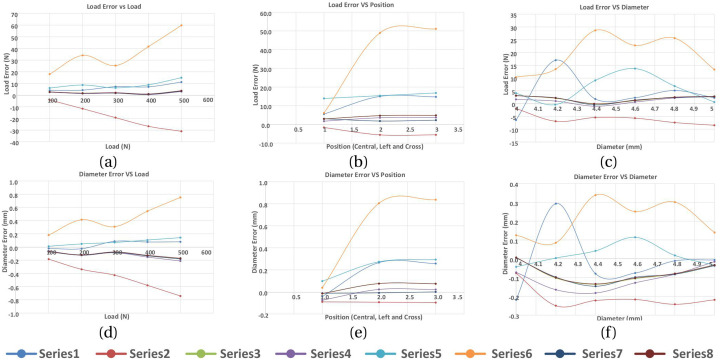
Load and tendon diameter errors in response to varying the tendon load, position and diameter. Series refer to Table 3. **(A)** Load error vs LOAD, **(B)** Load error vs position, **(C)** Load error vs diameter, **(D)** Diameter error vs load, **(E)** Diameter error vs position, **(F)** Diameter error vs diameter.

A series of model analyses was undertaken to assess the accuracy of the BT in measuring tendon force for variations in load, tendon size and position (Table 3 reports a subset of these). Several gauge locations were tested as per Table 1; for each location the tendon size and position were also varied, but only listed for one gauge location (Table 1, case 3) for brevity, as this was the only set of gauge placements of those tested which gave results which were satisfactory for the tendon located centrally and in line with the buckle. All other locations (Table 1, cases 1,2,4,5) gave errors in load (Table 3). Four of the six gauges are placed symmetrically about *X* and *Y*-axes (with the remaining two placed along the Y-axis in line with the central rod), and the modelled strains were in broad agreement with those expected from cantilever loading produced by the tendon. Strains in the X direction for each of the five gauge positions of Table 1 are shown in Table 4, for 500N tendon force and 4.4 mm tendon diameter. The strains at the outer gauge locations (sensors 1,2,5,6, Table 1) were in general lowest at the outer bearings and increased towards the centre, as shown by the strain gradient plot of Figure 6. For the one gauge location which gave satisfactory results for gauge locations (Table 1, case 3), the results for variation in tendon size and position were also satisfactory (within 2N error); Table 3, series 4. There was no change in these results when the other two strain combinations (Table 2 rows 1,3) were used; Table 3, series 7,8. For each of the eight series combinations of Table 3, the errors in predicted load and tendon diameter are shown in Figure 8, for variations in load, tendon diameter and position. Series 3,4,7,8 show best overall performance, all having X = 3 mm.

**TABLE 3:** Strain gauge coordinates, considered cases and resulting errors (load, N and tendon diameter, mm) for each gauge coordinate position.

Series	Location of sensors - cases (Table 1)	Strain combination (Table 2)	Position of the tendon	Average load error (N)	SD of load error	Average position error (mm)	SD of position error
1	1	2	central positions only	7	3	0.0	0.1
2	2	2	central positions only	−19	11	−0.5	0.2
3	3	2	central positions only	2	1	−0.1	0.0
4	3	2	central, left and cross positions	2	1	−0.1	0.1
5	4	2	central positions only	9	4	0.1	0.1
6	5	2	central positions only	36	16	0.4	0.2
7	3	1	central positions only	2	1	−0.1	0.0
8	3	3	central positions only	2	1	−0.1	0.0

SD: Standard Deviation

**TABLE 4:** X strains for 500N and 4.4 mm diameter tendon, for the 5 cases of Table 1.

Table 1 cases	D	E	X strain	X strain
	X,Y (mm)	X,Y (mm)		
1	1.9; 2.5	0; 2.5	−3.55E-05	−3.67E-05
2	1.9; 3.0	0; 3.0	−3.34E-05	−3.13E-05
3	2.9; 3.0	0; 3.0	−2.19E-05	−3.13E-05
4	4.1; 2.5	0; 2.5	2.96E-06	−3.67E-05
5	4.1; 3.0	0; 3.0	1.13E-05	−3.13E-05

## 4 Discussion

In this study, a prototype buckle transducer instrumented with several strain gauges has been FE modelled with loads applied directly to the end bearing surfaces and central rod, upon which the tendon would bear. Combinations of axial and shear forces were applied according to the angle of passage with which the tendon would pass through the transducer. Instead of modelling the morphology of a real tendon, we recognised that this would be true of only one tendon, and so we instead applied a distributed load to each bearing, and varied the centre and distribution of these loads to observe changes in the predicted tendon tension resulting from a regression model. Within a limited range of tendon diameters (3–5 mm), and corresponding central rod sizes to maintain an angle of passage which gave suitably large strains whilst reducing the tendon path length, strains were computed at several sites on the baseplate and used in linear regressions to determine the sensitivities to change in load and tendon thickness. The resulting load errors when varying load, tendon thickness and position were found to be acceptably small for a limited range of gauge positions around 3 mm from the baseplate centre in *X* and *Y* directions.

From the simple mathematical analysis presented earlier (Equations 10a-d, [Disp-formula e10a]), a significantly different magnitude of strain is required between central and outer gauges for the unknowns (T, α , β ) to be determined. Our results show that this is only realised for gauge locations around X = 3 mm (Table 4). For X = 2 mm the strains are higher but more similar to X = 0; for X = 4 mm the strains are opposite in sign and becoming too low to enable sufficient load resolution in a practical transducer. This highlights the importance of choosing appropriate gauge locations for the method to be able to yield satisfactory load errors irrespective of changes in tendon position and size (within the constraints of the BT). In a practical transducer the strain gauges occupy a finite area of the baseplate, thus averaging strains across the gauge grid; there is also less room for siting gauges appropriately, although the findings of this study would allow for suitable gauge sites practically. Moreover, these gauge locations are within regions of slowly changing strain fields, allowing for some immunity to positional errors.

The design of our BT attempted to allow for the inclusion of a cavity for electronics for *in vivo* measurement and telemetry. This necessarily stiffened the structure, such that the developed strains were low, Table 4, which would require the use of semiconductor strain gauges to achieve adequate load sensitivity. Future designs could aim to reduce this stiffness, minimising especially sidewall height which stiffens the cantilever action. Further refinement could include improved tendon contact simulation or even the inclusion of the tendon itself rather than its contacts with the transducer.

One aspect of the design which we did not explicitly test here is the ability of the transducer to determine loads over a range of angles of passage through the device, although the theory supports this. The next design iteration will include this.

We have shown here that an approach using multiple strain gauges is able to determine the tendon load independent of a certain range of tendon thickness and position in the buckle. This is shown theoretically by the mathematical analysis presented, although in a practical device, as in this FE study, the sensitivities would be found by regression.

## 5 Conclusion

This study introduces a miniaturized modular BT that offers adjustability to accommodate various Flexor Carpi Radialis tendon sizes. The initial hypothesis posits that the BT can be effectively calibrated regardless of tendon thickness and tension load. To explore this, the buckle was subjected to mathematical modeling and subsequent Solidworks simulations. By applying different forces to the buckle with varying bearing sizes, strain values were recorded across different cases. A sequence of regression analyses was devised to achieve two main objectives: I) determine the optimal location for installing gauge sensors on the buckle’s baseplate, and II) assess the model’s accuracy in calibrating the buckle while considering tendon position and tension load independence. The most favorable outcomes were achieved when the gauges were positioned with a maximum separation distance, avoiding proximity to the baseplate edges. The refined modular design of the BT indicates its potential readiness for the pilot study phase, involving its manufacturing and testing with Series 4 configuration for the installation of strain gauges. This particular configuration has demonstrated promising outcomes, notably in its capacity to facilitate the accurate determination of tendon tension while remaining unaffected by variations in local thickness. Our initial hypothesis was that the BT could be calibrated regardless of tendon thickness, placement and tension load. While the design shows promise in achieving this independence, variations in the angle of passage (AoP) within the BT were not adequately explored. As a result, further research is required to test for variation in AoP across different rod sizes and assess the accuracy of the transducer under these conditions. Subsequent phases of this research involve constructing a prototype BT for testing with human FCR tendons, aiming to compare real-world results against those attained through simulation. Furthermore, future possibilities entail *in vivo* device implantation to characterize forearm spasticity.

## Data Availability

The raw data supporting the conclusions of this article will be made available by the authors, without undue reservation.
